# Innate Immune Response of Human Embryonic Stem Cell-Derived Fibroblasts and Mesenchymal Stem Cells to Periodontopathogens

**DOI:** 10.1155/2016/8905365

**Published:** 2016-08-25

**Authors:** Gopu Sriram, Vaishali Prakash Natu, Intekhab Islam, Xin Fu, Chaminda Jayampath Seneviratne, Kai Soo Tan, Tong Cao

**Affiliations:** ^1^Discipline of Oral Sciences, Faculty of Dentistry, National University of Singapore, Singapore; ^2^Institute of Medical Biology, Agency for Science, Technology and Research (A^*^STAR), Singapore; ^3^Discipline of Oral and Maxillofacial Surgery, Faculty of Dentistry, National University of Singapore, Singapore; ^4^Research Center of Plastic Surgery Hospital, Chinese Academy of Medical Sciences, Peking Union Medical College, Beijing, China; ^5^NUS Graduate School for Integrative Sciences and Engineering, Singapore; ^6^Tissue Engineering Program, Life Sciences Institute, National University of Singapore, Singapore

## Abstract

Periodontitis involves complex interplay of bacteria and host immune response resulting in destruction of supporting tissues of the tooth. Toll-like receptors (TLRs) play a role in recognizing microbial pathogens and eliciting an innate immune response. Recently, the potential application of multipotent stem cells and pluripotent stem cells including human embryonic stem cells (hESCs) in periodontal regenerative therapy has been proposed. However, little is known about the impact of periodontopathogens on hESC-derived progenies. This study investigates the effects of heat-killed periodontopathogens, namely,* Porphyromonas gingivalis* and* Aggregatibacter actinomycetemcomitans*, on TLR and cytokine expression profile of hESC-derived progenies, namely, fibroblasts (hESC-Fib) and mesenchymal stem cells (hESC-MSCs). Additionally, the serotype-dependent effect of* A. actinomycetemcomitans* on hESC-derived progenies was explored. Both hESC-Fib and hESC-MSCs constitutively expressed* TLR-2* and* TLR-4*. hESC-Fib upon exposure to periodontopathogens displayed upregulation of TLRs and release of cytokines (IL-1*β*, IL-6, and IL-8). In contrast, hESC-MSCs were largely nonresponsive to bacterial challenge, especially in terms of cytokine production. Further, exposure of hESC-Fib to* A. actinomycetemcomitans* serotype c was associated with higher IL-8 production than serotype b. In contrast, the hESC-MSCs displayed no serotype-dependent response. Differential response of the two hESC progenies implies a phenotype-dependent response to periodontopathogens and supports the concept of immunomodulatory properties of MSCs.

## 1. Introduction

Periodontitis is a chronic inflammatory disease of the tooth supporting tissues which is accompanied by tissue destruction, weakening of tooth support, and eventually loss of tooth [1, 2]. It involves complex interplay of bacteria and host immune responses that ultimately lead to progressive destruction of the periodontium [3, 4]. Periodontopathic Gram-negative bacteria including* Porphyromonas gingivalis* (*P. gingivalis*) and* Aggregatibacter actinomycetemcomitans* (*A. actinomycetemcomitans*) (previously known as* Actinobacillus actinomycetemcomitans*) have been strongly implicated in periodontitis [5, 6]. Various components of these periodontopathogens, such as lipopolysaccharides (LPS), lipoproteins, and fimbriae, interact with the host through various pattern-recognition receptors [7, 8]. Toll-like receptors (TLRs) are a family of pattern-recognition receptors evolved to detect various components of pathogens and have various downstream effects [9]. This involves activation of intracellular signaling cascade which stimulates transcription factors which finally leads to inflammatory cytokine expression, activation of immune cells, migration of leukocytes, and osteoclastogenesis [10]. Among the TLRs, TLR-2 and TLR-4 function as the principal innate sensors for cell wall components of Gram-negative bacteria in mammals and are considered crucial in the progress of periodontitis [11, 12]. TLR-2 and TLR-4 stimulation leads to activation of proinflammatory cytokines and chemokines which initiates the inflammatory process [13, 14]. Cell wall components of* P. gingivalis* and* A. actinomycetemcomitans* stimulate, via TLR-2 and TLR-4, the production of proinflammatory cytokines like interleukins IL-1*β* and IL-6 which can induce production of matrix metalloproteinases and mediate alveolar bone resorption [15].

The goal of periodontal therapy is to halt the disease process and promote regeneration of the lost periodontal tissues. Currently available treatment modalities result in improved clinical outcomes; however, they are insufficient to achieve complete periodontal regeneration [16]. Currently, various biomaterial and/or cell-based approaches for formation and regeneration of periodontal tissues are explored (excellently reviewed elsewhere [16–18]). Recently, multipotent stem cells derived from various orodental tissues and pluripotent stem cells including human embryonic stem cells (hESCs) have been proposed as a promising source of cells for such cell-based periodontal regenerative therapies [19–21]. Previous studies on miniature pigs have shown that local cellular therapy with autologous and allogeneic periodontal ligament stem cells (PDL-SCs) is associated with improved periodontal tissue regeneration [22, 23]. MSCs are proposed to possess immunomodulatory properties through secretion of a host of soluble factors and/or direct cell-cell contact. The immunomodulatory properties of MSCs might offer a promising approach for periodontal regeneration. However, precise mechanisms are poorly understood, which limit the clinical application of MSCs.

hESCs are a potential source of stem cells due to their ability to self-renew and differentiate into virtually any cell type of the human body [24, 25]. Further, hESCs could be utilized to generate unlimited numbers of healthy and functional fibroblasts and mesenchymal stem cells (MSCs) that lack prior exposure to periodontopathogens. Recently, we [26–30] have developed methods to differentiate hESCs to fibroblasts and mesenchymal stem cells (MSCs). However, the potential impact of periodontopathogens on these hESC-derived progenies remains poorly understood. Until now, little is known about the ability of hESC-derived progenies to express TLRs for sensing bacterial pathogens and their influence on cytokine secretory profile. Thus, a better understanding of the effects of exposure to periodontopathogens on TLR and cytokine expression by hESC-derived progenies could be crucial for their successful application.

In this study, we sought to comparatively investigate the effects of heat-killed* P. gingivalis* and* A. actinomycetemcomitans* on TLR and cytokine expression profile of human periodontal ligament fibroblasts (hPLFs) and hESC-derived progenies, namely, fibroblasts (hESC-Fib) and MSCs (hESC-MSCs). Further, we investigated the influence of* A. actinomycetemcomitans* serotypes on TLR and cytokine expression profiles in order to explore strain-dependent effect within the same bacterial species.

## 2. Materials and Methods

### 2.1. Culture of hESCs

In this study, H1-hESCs (WiCell Research Institute, Madison, WI) were cultured on mitomycin-C inactivated-murine embryonic fibroblasts (MEFs) using hESC medium as described previously [31, 32]. Briefly, the hESC medium consisted of Dulbecco's modified Eagle's medium (DMEM)/Ham's F12 (1 : 1) supplemented with 20% Knockout Serum Replacement (KO-SR, Gibco), 1% (vol/vol) nonessential amino acids, 1 mM L-glutamine (Gibco), 4 ng/mL basic fibroblast growth factor (bFGF, Invitrogen), and 0.1 mM *β*-mercaptoethanol (Sigma). Media were changed every other day and passaged every 6-7 days using 1 mg/mL collagenase type IV (Gibco) for 5 minutes, followed by manual dissociation to small clumps and seeding onto MEF-seeded plates.

### 2.2. Differentiation of hESCs to Fibroblast-Like Cells

Fibroblast-like cells generated from hESCs are termed hESC-Fib (hESC-derived fibroblast-like cells) and were generated by methods described previously [26, 27]. Confluent H1-hESC colonies were detached from the feeder layer using collagenase type IV (1 mg/mL). Large cell aggregates were broken up and replated in EB media [DMEM/F12 supplemented with 20% KO-SR, 1% (vol/vol) nonessential amino acids, 1 mM L-glutamine, and 0.1 mM *β*-mercaptoethanol] in ultralow-attachment plates (Corning). After 24 hours, the suspended hESC clumps formed free-floating spheroidal aggregates or embryoid bodies (EBs). The culture medium was changed every 2 days. After 5 days, EBs were harvested and plated onto gelatin-coated plates in fibroblast medium [DMEM (high glucose) supplemented with 10% fetal bovine serum (FBS, Biowest), 1 mM L-glutamine, and penicillin/streptomycin (100 U/mL and 100 mg/mL, resp.)]. After 15 days, the EB outgrowths were subcultured using TrypLE*™* Express (Gibco). After three such subcultures, the EB outgrowths attained homogenous population of spindle-shaped cells. These cells are termed hESC-Fib and were designated as passage 0. Passages 5–8 hESC-Fib were used in all subsequent experiments.

### 2.3. Differentiation of hESCs to MSC-Like Cells

MSC-like cells generated from hESCs are termed hESC-MSCs and were generated by methods described previously [28, 30]. hESCs were differentiated to hESC-MSCs using a two-step process involving EB formation, followed by outgrowth of EBs over gelatin-coated plates as described above. Briefly, H1-hESCs colonies were dissociated into small clumps after 15–20 minutes of incubation with 1 mg/mL collagenase type IV and then transferred to ultralow-attachment plates in EB media. After 7 days, EBs were harvested and plated onto gelatin-coated plates in MSC induction medium [DMEM (low glucose) supplemented with 10% FBS, 1 mM L-glutamine, and 1% penicillin/streptomycin]. After 2 weeks, the EB outgrowths were subcultured using TrypLE Express. After the 2nd passage, the cells were maintained in MSC differentiation medium (PromoCell). The differentiated hESCs attained homogenous population of spindle-shaped cells. These are termed hESC-MSCs and were designated as passage 0. Passages 4–8 hESC-MSCs were used in all subsequent experiments.

### 2.4. Culture of Human Periodontal Ligament Fibroblasts

Pooled primary human periodontal ligament fibroblasts (hPLFs) were obtained from a commercial source (ScienCell Research Laboratories) and were cultured as previously described [33, 34]. Briefly, the hPLFs were cultured in fibroblast medium [DMEM (high glucose) supplemented with 10% fetal bovine serum, 1 mM L-glutamine, and 1% penicillin/streptomycin]. Passages 4–6 hPLFs were used in all subsequent experiments.

### 2.5. Culture of Periodontopathogens

*P. gingivalis* (ATCC W50),* A. actinomycetemcomitans* serotype b (ATCC 700685, JP2 clone), and* A. actinomycetemcomitans* serotype c (ATCC 33384) were obtained from American Type Culture Collection (ATCC).* P. gingivalis* was cultured on trypticase soy agar (TSA) with 5% sheep blood agar (Oxoid) and incubated at 37°C in an anaerobic chamber (Don Whitley Scientific) while* A. actinomycetemcomitans* was cultured on brain heart infusion (BHI) agar (Acumedia) and incubated at 37°C with 5% CO_2_. Broth cultures of* P. gingivalis* and* A. actinomycetemcomitans* were prepared by inoculating an isolated bacterial colony into BHI broth supplemented with yeast extract (Acumedia), hemin (Sigma), and vitamin K (Sigma) as previously described [35] and incubated as described above for 24 hours. Bacterial pellet was washed twice with sterile phosphate buffered saline (PBS) before resuspending in sterile water. Heat-killed bacteria (HKB) were prepared by heating at 60°C for 30 minutes and aliquots were stored at −20°C.

### 2.6. Bacterial Challenge Assays

The cells (hESC-Fib, hESC-MSCs, and hPLFs) were seeded in 6/96-well plates and grown in respective medium for 2 days till they reached a subconfluent stage. Each well of fibroblasts was subjected to bacterial challenge (*P. gingivalis* and two different serotypes of* A. actinomycetemcomitans*) with a multiplicity of infection (MOI) of 1 : 100 (cells : bacteria) and incubated at 37°C with 5% CO_2_ for 24 to 48 hours. Culture wells without bacterial challenge were used as control. Morphology of the cells was checked for abnormalities or cell death using phase-contrast microscopy. The metabolic activity of the cells was assayed using MTS assay after 24 and 48 hours of bacterial challenge. For transcript and protein analysis, the cells were challenged with bacteria for 24 hours. The culture supernatants were harvested and stored at −80°C for protein assays. Following this, the cells were lysed and used for RNA extraction using RNeasy mini kit (Qiagen) as per the manufacturer's instructions.

### 2.7. Phenotyping of hESC-MSCs

The phenotype of hESC-MSCs was characterized using flow cytometry and the following monoclonal antibodies: anti-CD31-APC (Miltenyi Biotec), anti-CD44-FITC (BD Pharmingen), anti-CD45-FITC (BD Pharmingen), anti-CD73-APC (Miltenyi Biotec), anti-CD90-FITC (BD Pharmingen), anti-CD105-PE (eBioscience), HLA-ABC-APC (BD Pharmingen), and HLA-DR-FITC (BD Pharmingen). Briefly, the cells were dissociated and suspended in FACS buffer (1x PBS/0.5% BSA) and nonspecific binding blocked with FcR blocking agent (Miltenyi Biotec) for 10 minutes at 4°C. For labeling cell surface antigens, the cells were incubated with the abovementioned fluorescent conjugated antibodies for 10 minutes at 4°C. After antibody labeling, data was acquired using Dako Cytomation CyAn ADP and analyzed using FlowJo v7.6.5 (Tree Star).

### 2.8. Real-Time Reverse Transcriptase Polymerase Chain Reaction (Real-Time RT-PCR)

Harvested mRNA was reverse transcribed using iScript*™* cDNA synthesis kit (Bio-Rad) according to the manufacturer's instructions. Real-time RT-PCR was performed in triplicate using Fast SYBR Green PCR master mix (Applied Biosystems) and processed on StepOnePlus Real-Time PCR System (Applied Biosystems) as per the manufacturer's instructions. Briefly, after a 20 sec activation step at 95°C, 40 cycles of a two-step PCR were run which consisted of denaturation at 95°C for 3 sec, followed by an annealing and extension step at 60°C for 30 sec. Further, the PCR products were subjected to melt curve analysis to exclude the generation of nonspecific PCR products. The expression levels of target genes were quantified by normalization against corresponding endogenous reference *β-ACTIN* and expressed as fold change relative to respective control samples using the ΔΔC_T_ method. For presenting the constitutive expression of respective genes, relative quantity was calculated with *β-ACTIN* as a reference, by using the formula 2^(−[C_T GENE_−C_T *β*-*ACTIN*_])^. Details of primer sequences used in this study are presented in Table 1. The results are presented as mean ± standard deviations of three independent experiments.

### 2.9. Cytokine Analysis

Enzyme linked immunosorbent assays (ELISA) were performed to determine the protein levels of IL-6 and IL-8 using the respective ELISA kits (R&D Systems) according to the manufacturer's protocol. Prior to the ELISA, the culture supernatants were centrifuged to pellet the cellular and bacterial remains. The supernatants were harvested, diluted using dilution buffer supplied with the kits, and used for the protein estimation. The results are presented as mean ± standard deviations of three independent experiments.

### 2.10. MTS Assay

This assay was used to determine the metabolic activity of the cells after addition of bacteria. 96-well plates were seeded with the cells and cultured in respective medium for 24 hours. Subsequently, bacteria were added to the wells at an MOI of 1 : 100 (cells : bacteria). Wells without the bacteria served as control. After 24 and 48 hours of bacterial challenge, media were removed, washed with PBS, and replaced with MTS [(3-(4,5-dimethylthiazol-2-yl)-5-(3-carboxymethoxyphenyl)-2-(4-sulfophenyl)-2H-tetrazolium)] reagent (Promega) and incubated at 37°C as per the manufacturer's instructions. Following incubation for 2 hours, the absorbance was measured at 490 nm using a microplate reader (Tecan).

### 2.11. Statistical Analysis

The results are presented as mean ± standard deviation of three experiments. Statistical differences were evaluated by a two-tailed *t*-test or one-way ANOVA followed by Tukey's post hoc test. *p* values <0.05 were considered statistically significant.

## 3. Results

### 3.1. Derivation and Characterization of hESC-Fib

**Figure 1:**
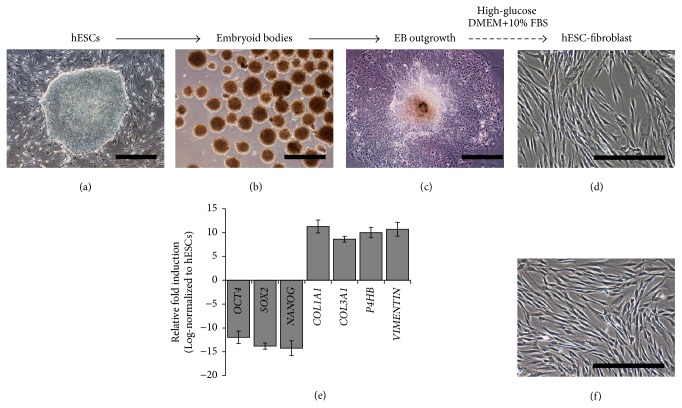
Differentiation of hESCs to hESC-Fib. (a–d) The photomicrographs demonstrate the differentiation of hESCs to embryoid bodies, embryoid body outgrowth, and hESC-Fib under high-glucose differentiation conditions. (e) Characterization of hESC-Fib by real-time RT-PCR for pluripotency (OCT4, SOX2, and NANOG) and fibroblast-related transcripts (*COL1A1*,* COL3A1*,* P4Hβ*, and* VIMENTIN*). (f) Photomicrograph shows the spindle-shaped hPLFs. Scale bar: 500 *µ*m.

To derive fibroblast-like cells from hESCs, H1-hESCs were differentiated through an EB outgrowth method under high-glucose culture conditions as previously described by us [26, 27, 32]. Undifferentiated hESCs exhibited compact colony morphology with defined borders (Figure 1(a)). After 24 hours of suspension culture, aggregates of hESC colonies formed EBs (Figure 1(b)). Adherent culture of EBs over gelatin-coated plates resulted in migration of fibroblast-like cells within 48 h of attachment. The migrating cells were called EB outgrowths, which proliferated gradually and reached confluence after 2 weeks of culture (Figure 1(c)). After 3 passages, the spindle-shaped fibroblast-like cells attained homogeneous morphology and were termed hESC-Fib (Figure 1(d)). Real-time RT-PCR analysis of the hESC-Fib demonstrated the downregulation of pluripotency markers (*OCT4*,* SOX2*, and* NANOG*) and upregulation of fibroblast-related markers (*COL1A1*,* COL3A1*,* P4Hβ*, and* VIMENTIN*) (Figure 1(e)). The hESC-Fib had spindle-shaped morphology similar to that of hPLFs (Figure 1(f)).

### 3.2. Differentiation and Characterization of hESC-MSCs

**Figure 2:**
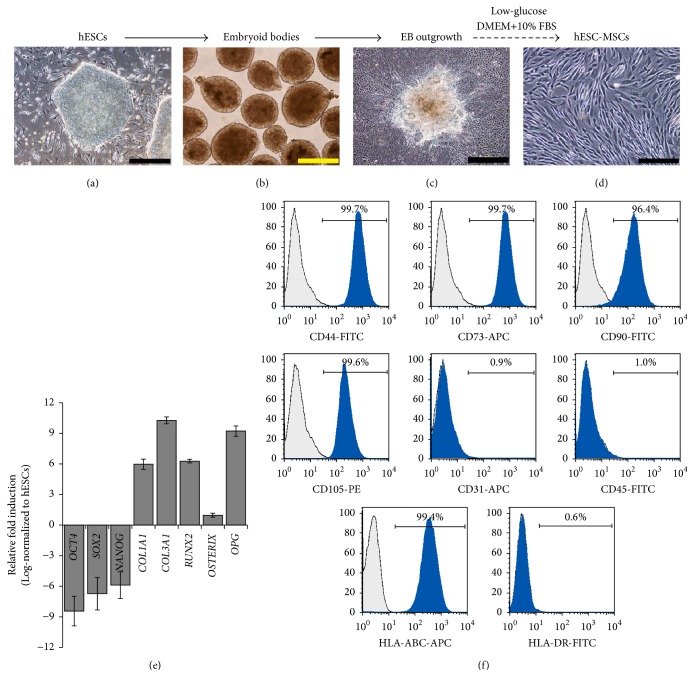
Differentiation of hESCs to hESC-MSCs. (a–d) The photomicrographs demonstrate the differentiation of hESCs to hESC-MSCs through embryoid bodies and embryoid body outgrowth under low-glucose differentiation conditions. (e) Characterization of hESC-MSCs by real-time RT-PCR for pluripotency (OCT4, SOX2, and NANOG) and mesenchymal (*COL1A1*,* COL3A1*) and osteogenic (*RUNX2*,* OSTERIX*, and* OPG*) lineage associated transcripts. (f) Flow cytometry characterization of hESC-MSCs for expression of surface markers. Scale bar in (a) and (c): 500 *µ*m. Scale bar in (b) and (d): 200 *µ*m.

To generate MSC-like cells from hESCs, H1-hESCs were differentiated through a two-stage EB outgrowth method under low-glucose conditions as previously described [28, 30]. Briefly, EBs were generated by suspension culture of aggregates of hESCs (Figures 2(a) and 2(b)). After 10 days of differentiation under suspension culture, EBs were plated on gelatin-coated plates (Figure 2(c)). Upon adherent culture under low-glucose culture conditions, spindle-shaped cells migrated out from the EBs. These spindle-shaped EB outgrowth cells were subcultured under low-glucose conditions for 2 passages before the cells attained homogeneous morphology and were termed hESC-MSCs (Figure 2(d)). The phenotype and multilineage differentiation ability of these hESC-MSCs have been characterized and published earlier [28, 30, 36]. Real-time RT-PCR analysis of the hESC-MSCs demonstrated the downregulation of transcripts related to pluripotency (*OCT4*,* SOX2*, and* NANOG*) and upregulation of mesenchymal (*COL1A1*,* COL3A1*) and osteogenic (*RUNX2*,* OSTERIX*, and* OPG*) transcripts (Figure 2(e)). Further, flow cytometry analysis markers of MSCs demonstrated expression of MSC-associated surface markers CD44 (99.7%), CD73 (99.7%), CD90 (96.4%), and CD105 (99.6%) and were negative for CD31 (1%) and CD45 (1%) (Figure 2(f)). Further, majority of hESC-MSCs (>99%) displayed the expression of HLA class I molecule HLA-ABC at high levels but lacked the expression of HLA class II molecule HLA-DR (1%) (Figure 2(f)).

### 3.3. Bacterial Challenge Does Not Affect Cellular Morphology and Viability

**Figure 3:**
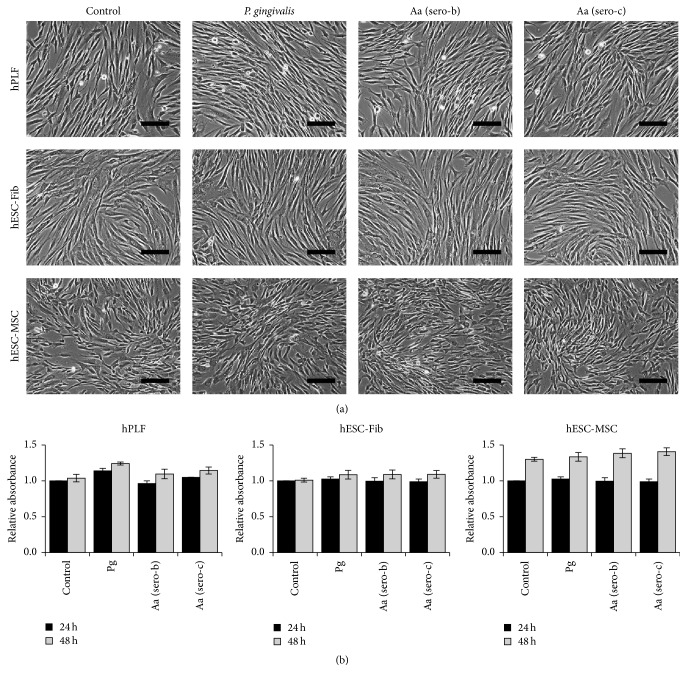
Viability and proliferation of hPLFs, hESC-Fib, and hESC-MSCs. (a) Morphology of control and treated groups after 48 hours of bacterial challenge. (b) Viability and proliferation of cells assessed by MTS assay after exposure to* P. gingivalis* (Pg) and two different serotypes of* A. actinomycetemcomitans* (Aa serotypes b and c) for 24 and 48 hours. Scale bar: 200 *µ*m.

Cellular morphology, viability, and proliferation of hPLFs, hESC-Fib, and hESC-MSCs were assessed by phase-contrast microscopy and MTS assay after 24 and 48 hours of bacterial challenge. Fibroblast cultures devoid of bacterial challenge were used as negative control. The morphology of all the three cell types was not affected by exposure to* P. gingivalis* and* A. actinomycetemcomitans* (Figure 3(a)). Based on MTS assay, hPLFs, hESC-Fib, and hESC-MSCs were viable and proliferative after 24 and 48 hours of challenge with all three strains of bacteria (Figure 3(b)).

### 3.4. Constitutive Expression of TLRs and Cytokines in Fibroblasts and MSCs

**Figure 4:**
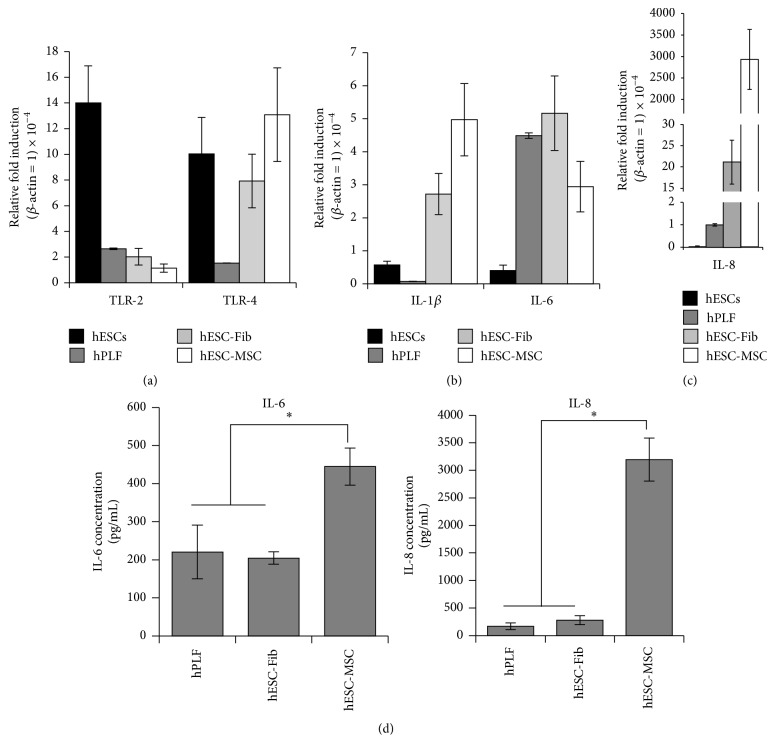
Basal expression profiles of TLR and cytokines in hESCs, hPLFs, hESC-Fib, and hESC-MSCs. Basal expression profiles of (a) TLR-2 and TLR-4 and (b, c) IL-1*β*, IL-6, and IL-8 among hESCs, hPLFs, hESC-Fib, and hESC-MSCs as analyzed by real-time RT-PCR. The relative fold induction is relative to the respective transcript levels of *β*-actin. The *y*-axis in (c) is broken to enable visualization of the relative expression levels of IL-8 among the different cell types. (d) Basal levels of cytokine production in the culture supernatants (assayed using ELISA) from hPLFs, hESC-Fib, and hESC-MSCs. The production of IL-6 and IL-8 by hESCs was below detection limits. Values represent the means ± SD of three experiments (^*∗*^ *p* < 0.05).

The expression profiles of TLRs in hESCs, fibroblasts, and MSCs were analyzed at mRNA level by real-time RT-PCR, while the profiles of cytokine expression were analyzed at transcript and protein levels using real-time RT-PCR and ELISA. Real-time RT-PCR analysis demonstrated that hPLFs, hESCs, hESC-Fib, and hESC-MSCs constitutively expressed* TLR-2* and* TLR-4* (Figure 4(a)). Interestingly, hESCs expressed higher levels of* TLR-2* and* TLR-4*. The hESC-derived progenies (hESC-Fib and hESC-MSCs) expressed relatively higher levels of* TLR-4* than* TLR-2*.

Prior to investigation into the effect of bacterial challenge, basal expression levels of cytokines (*IL-1β*,* IL-6*, and* IL-8*) in hESCs, hPLFs, hESC-Fib, and hESC-MSCs were quantitatively analyzed (Figure 4). Real-time RT-PCR analysis demonstrated that hESCs and hPLFs expressed low levels of all the three cytokines. hESC-Fib expressed low levels of* IL-1β* and* IL-6* and moderate levels of* IL-8*. On the contrary, hESC-MSCs expressed low levels of* IL-1β* and* IL-6* similar to hESC-Fib but extremely high levels of* IL-8* (Figures 4(b) and 4(c)). Absolute quantification of IL-6 and IL-8 secretion using ELISA shows significantly high levels of IL-6 and IL-8 secretion by hESC-MSCs compared to hPLFs and hESC-Fib, while there was no significant difference in the cytokine levels secreted by hPLFs and hESC-Fib (Figure 4(d)).

### 3.5. Influence of Bacterial Challenge on TLR Expression in Fibroblasts and MSCs

**Figure 5:**
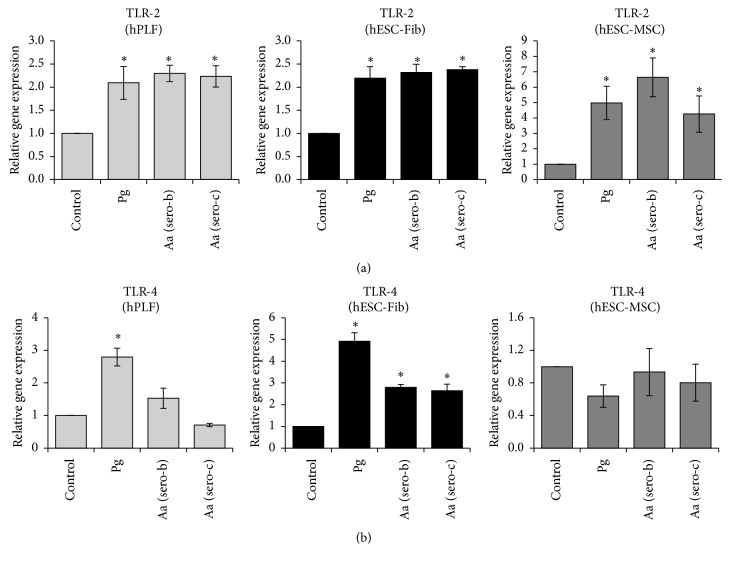
Transcript expression profiles of TLR-2 (a) and TLR-4 (b) among hPLFs, hESC-Fib, and hESC-MSCs in response to exposure to* P. gingivalis* (Pg) and two different serotypes of* A. actinomycetemcomitans* (Aa serotypes b and c). The transcript levels were normalized to the respective *β*-actin levels and to the untreated control sample. Values represent the means ± SD of three experiments (^*∗*^ *p* < 0.05 versus untreated control).

To investigate the effect of bacterial challenge on TLR expression, hPLFs, hESC-Fib, and hESC-MSCs were exposed to heat-killed* P. gingivalis* and two different serotypes of* A. actinomycetemcomitans* at an MOI of 1 : 100 for 24 hours. Unchallenged cells were used as negative control. Exposure to the three different Gram-negative bacteria induced significant upregulation of* TLR-2* expression in all the three cell types (Figure 5(a)). The fibroblast populations (hPLFs and hESC-Fib) exhibited ~2-fold increase in* TLR-2* expression upon challenge with all the three Gram-negative bacteria, while the hESC-MSCs exhibited 5–7-fold increase in* TLR-2* expression under similar conditions. Further, the three cell types exhibited differential* TLR-4* expression after bacterial challenge. hPLFs exhibited significant upregulation of* TLR-4* expression upon exposure to* P. gingivalis* only, while in hESC-Fib all the three Gram-negative bacteria induced* TLR-4* upregulation. However, hESC-MSCs displayed no significant change in* TLR-4* expression upon exposure to all the three Gram-negative bacteria (Figure 5(b)).

### 3.6. Bacterial Challenge Induces Cytokine Expression in Fibroblasts, but Not in hESC-MSCs

**Figure 6:**
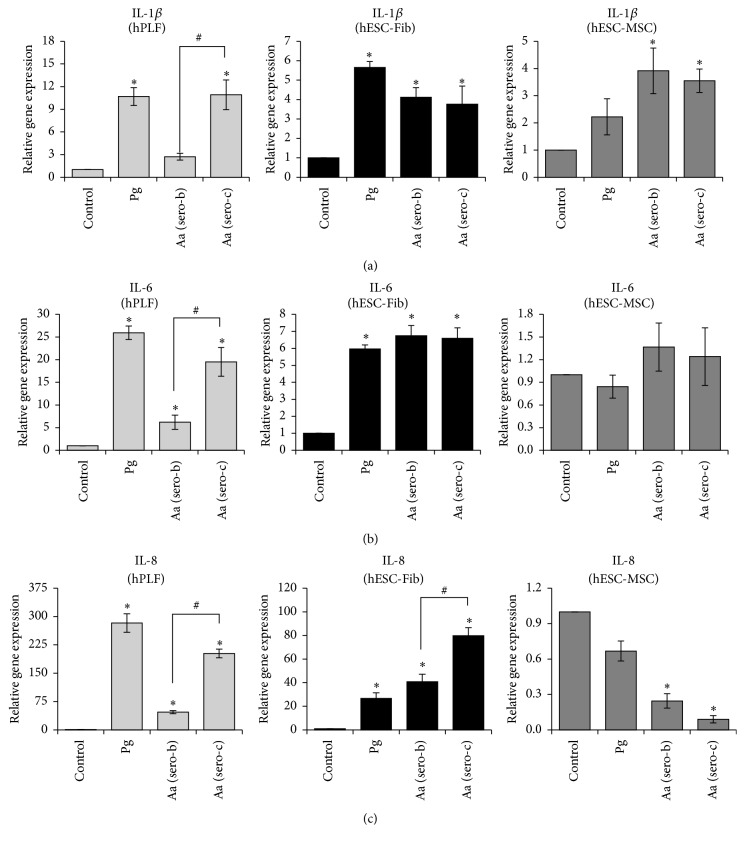
Transcript expression profiles of cytokines IL-1*β* (a), IL-6 (b), and IL-8 (c) among hPLFs, hESC-Fib, and hESC-MSCs in response to* P. gingivalis* (Pg) and two different serotypes of* A. actinomycetemcomitans* (Aa serotypes b and c). The transcript levels were normalized to the respective *β*-actin levels and to the untreated control sample. Values represent the means ± SD of three experiments (^*∗*^ *p* < 0.05 versus untreated control; ^#^ *p* < 0.05 versus different serotypes of* A. actinomycetemcomitans*).

**Figure 7:**
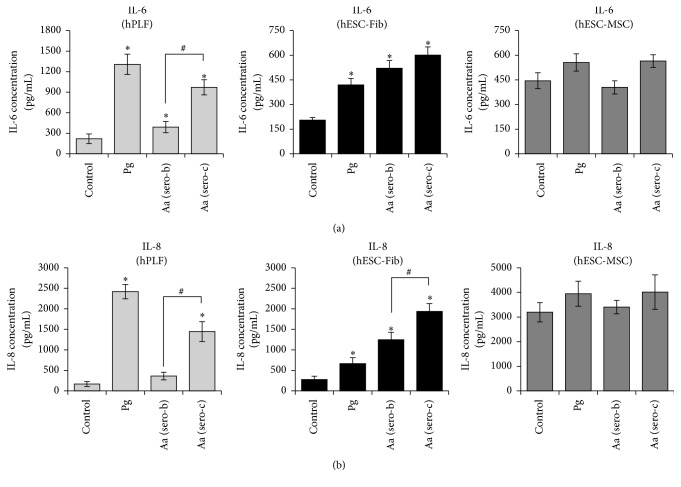
IL-6 (a) and IL-8 (b) production in the culture supernatants (assayed using ELISA) from hPLFs, hESC-Fib, and hESC-MSCs in response to exposure to* P. gingivalis* (Pg) and two different serotypes of* A. actinomycetemcomitans* (Aa serotypes b and c). Values represent the means ± SD of three experiments (^*∗*^ *p* < 0.05 versus untreated control; ^#^ *p* < 0.05 versus different serotypes of* A. actinomycetemcomitans*).

Exposure of hPLFs to Gram-negative bacteria resulted in significant upregulation of all the three cytokines (*IL-1β*,* IL-6*, and* IL-8*) (Figure 6). Estimation of the amount of cytokines released into the culture media also showed a significant increase in production of IL-6 and IL-8 upon exposure to the Gram-negative bacteria (Figure 7). Similarly, exposure of hESC-Fib to Gram-negative bacteria resulted in significant upregulation of* IL-1β*,* IL-6*, and* IL-8* and increased secretion of IL-6 and IL-8 (Figures 6 and 7). In contrast, exposure of hESC-MSCs to* P. gingivalis* had no significant effect on the expression of* IL-1β*,* IL-6*, and* IL-8*. Similarly, exposure of hESC-MSCs to* A. actinomycetemcomitans* induced significant upregulation of* IL-1β* but had no effect on* IL-6* and significantly downregulated* IL-8* expression at transcript levels (Figure 6). However, quantification of cytokine secretion levels showed that all the three Gram-negative bacteria had no significant effect on the secretion of IL-6 and IL-8 in hESC-MSCs (Figure 7). The discrepancy in transcript and protein levels might be due to the high constitutive expression of cytokines by the hESC-MSCs. Though the bacterial challenge of hESC-MSCs is associated with TLR-2 upregulation, there is no effect on production of cytokines studies. Further studies on other inflammatory and anti-inflammatory cytokines like TGF*β*, TNF-*α*, IL-10, and nitric oxide might shed more light on their response to bacterial challenge.

Overall, these results indicate the differential expression of cytokines upon exposure to Gram-negative bacteria depending on the cell type investigated. Further, the results highlight the distinct difference in the response of hESC-MSCs compared to the fibroblast phenotypes and perhaps support the immunomodulatory properties of MSCs.

### 3.7. Effect of* A. actinomycetemcomitans* Serotype on TLR and Cytokine Expression

The influence of* A. actinomycetemcomitans* serotypes on* TLR* expression was compared in order to investigate strain-dependent effect within the same bacterial species. The cells were challenged with* A. actinomycetemcomitans* serotype b (*Aa* ATCC 700685) or* A. actinomycetemcomitans* serotype c (*Aa* ATCC 33384). hPLFs and hESC-derived progenies displayed no significant difference between the two* Aa* strains in activating* TLR-2* and* TLR-4* expression (Figure 5).

Interestingly, there were striking differences among the two serotypes of* A. actinomycetemcomitans* in cytokine production.* Aa* serotype c induced significant upregulation of* IL-1β*,* IL-6*, and* IL-8* compared to* Aa* serotype b in hPLFs (Figure 6). Absolute quantification of cytokine production also revealed significantly higher production of IL-6 and IL-8 by hPLFs exposed to* Aa* (serotype c) (Figure 7). Similarly, exposure of hESC-Fib to* Aa serotype c* was associated with significant upregulation and production of IL-8 compared to Aa serotype b but had no differential effect on IL-1*β* and IL-6 expression (Figures 6 and 7). In contrast, the* A. actinomycetemcomitans* serotype-dependent variability in cytokine expression was not observed among hESC-MSCs. The discrepancy between hPLFs and hESC-Fib in response to the Aa serotypes might be due to various reasons. Considering the origin from hESCs, hESC-Fib might still be immature compared to hPLFs. Secondly, hPLFs could be primed to bacterial challenge* in vivo*, while hESC-Fib are a naïve population of fibroblasts without any prior exposure to bacteria.

These results indicate that* Aa* serotype c induces stronger cytokine expression among fibroblasts compared to* Aa* serotype b, while on the contrary hESC-MSCs are largely nonresponsive to both serotypes of* A. actinomycetemcomitans*. However, future studies on the effect on other downstream pathways need to be validated.

## 4. Discussion

In the present study, hESC-derived progenies (hESC-Fib and hESC-MSCs) were obtained through directed differentiation of hESCs. Then, we investigated the effects of heat-killed* P. gingivalis* and* A. actinomycetemcomitans* on cell viability, TLRs, and cytokine expression profile of human periodontal ligament fibroblasts (hPLFs) and hESC-derived progenies, namely, fibroblasts (hESC-Fib) and MSCs (hESC-MSCs). The impact of different periodontopathogens on hPLFs and hESC-derived progenies seems to be dependent on the cell type and, to a certain extent, the strain of the periodontopathogen itself.

Among the three cell types investigated, hESC-MSCs had distinctly different response to periodontopathogens. Particularly, hESC-MSCs displayed a low immunogenic profile after exposure to periodontopathogens, featuring absence of changes in the expression levels of* TLR-4* and cytokines (IL-6 and IL-8). Even though the hESC-MSCs expressed* TLR-2* and* TLR-4* constitutively, there was no change in TLR-inducible cytokines, indicating ineffective downstream signaling. Fu et al. demonstrated that hESC-MSCs share similar immunogenicity and immunoresponsive abilities like bone marrow-derived MSCs, but they exhibit differences in the expression of immunological markers and response to inflammatory cytokines suggesting that hESC-MSCs could be a potential candidate for stem cell therapy in inflammatory disorders [30].

Challenge of gingival and periodontal fibroblasts with putative periodontopathogens or their antigenic components has been reported to upregulate the immunoregulatory modulators such as IL-1*β*, IL-6, and IL-8 [37–39]. Dysregulated production of these immunoregulatory molecules in response to periodontopathogen exposure may result in excessive amplification of immune response and hence play a crucial role in periodontal tissue destruction [6, 40]. Data in the present study indicate that unchallenged hPLF, hESC-Fib, and hESC-MSCs maintained in culture for 24 hours are capable of secreting IL-1*β*, IL-6, and IL-8. Interestingly, unchallenged hPLFs and hESC-Fib secrete low levels of IL-6 and IL-8, while hESC-MSCs secrete high levels of both cytokines. The present study has shown that putative periodontopathogens can differentially alter the magnitude of cytokines production by hPLF, hESC-Fib, and hESC-MSCs. Upregulation of TLRs and cytokine production by hPLFs and hESC-Fib in response to* P. gingivalis* and* A. actinomycetemcomitans* exemplifies the ability of these fibroblasts to respond to and influence the outcome of the inflammatory response during the progression of periodontal disease. On the other hand, absence of cytokine response in hESC-MSCs indicates the differential response of these cells under bacterial challenge that could alter the progression of periodontal disease differently.* A. actinomycetemcomitans* has been associated with aggressive forms of periodontitis, and one possible mechanism whereby this pathogen could contribute to rapid destruction of periodontal tissues is by stimulating the fibroblasts to produce IL-6 and IL-8 [41]. Cytokines like IL-1*α*, IL-1*β*, IL-6, IL-8, and TNF-*α* have been documented to be involved in immune activation, increased cytotoxic activity, and cytokine-mediated osteoclastic bone resorption in aggressive forms of periodontitis [10, 42–44]. The absence of stimulatory effects on hESC-MSCs could positively influence the progression of aggressive periodontal disease. However, the relevance of high levels of constitutive expression of cytokines by hESC-MSCs and their role in cellular therapy warrants further investigation.

TLR and cytokine expression profiles of hESC-Fib and hESC-MSCs in response to periodontopathogens have not been described previously. Studies on hESC-derived progenies reported that undifferentiated hESCs, hESC-derived cardiomyocytes, and endothelial cells (ECs) were nonresponsive to bacterial challenge [45]. Further, ECs derived from primary adult or fetal vessels and from stem cells like blood progenitors and induced pluripotent stem cells were responsive to LPS. In contrast, the ECs derived from hESCs were not responsive [45, 46]. Though hESC-derived ECs were found to lack functional TLR-4, they were responsive to challenge with Gram-negative bacteria through NOD1 pathway [46]. Recent studies on hESC-derived keratinocytes have shown that these cells express TLRs and cytokines through activation of nuclear factor *κ*B (NF*κ*B) in response to exposure to* P. gingivalis* similar to a keratinocyte cell line [47]. We observed that hESCs, hESC-Fib, and hESC-MSCs constitutively expressed* TLR-2* and* TLR-4*. In spite of the origin from the same cell source, the expression of TLRs and cytokines in response to periodontopathogens is distinctly different among hESC-Fib and hESC-MSCs. Exposure to periodontopathogens resulted in upregulation of both* TLR-2* and* TLR-4* in hESC-Fib, but only* TLR-2* in hESC-MSCs. Further, bacterial challenge of hESC-Fib was associated with upregulation of all three cytokines (*IL-1β*,* IL-6*, and* IL-8*) investigated. On the other hand, bacterial challenge of hESC-MSCs was associated with upregulation of* IL-1β* only.

Considering the fact that both hESC-Fib and hESC-MSCs have been derived from the same cell source, the difference in TLR and cytokine expression profiles implied a phenotype-dependent response to periodontopathogens and supported the concept of immunomodulatory properties of MSCs upon bacterial challenge. Studies have shown that the immunomodulatory property of MSCs seems to depend on their origin, as differences between bone marrow, adipose tissue, and Wharton's jelly-derived MSCs were found recently [48, 49]. These studies demonstrated the resistance of Wharton's jelly-derived MSCs to bacterial challenge compared to other postnatal sources of MSCs. This difference could be attributed to the primitive nature of Wharton's jelly-derived MSCs compared to other postnatal MSCs [48, 50] and/or expression of nonfunctional TLRs [48]. Similarly, studies comparing PDL-SCs and bone marrow MSCs (BM-MSCs) have demonstrated that LPS and/or TNF-*α* differentially decreased the osteogenic differentiation ability of PDLSCs through TLR-4-mediated NF*κ*B [51, 52] and Wnt [53] signaling pathways. These results suggest a stronger immunomodulatory profile of BM-MSCs compared to PDL-SCs which might be due to pathological alterations caused by inflammatory insults on the latter. hESCs are one of the most primitive stem cells, and hence hESC-MSCs could also be of the most primitive state similar to WJ-MSCs. Bacterial challenge of hPLFs and hESC-Fib results in increased production of cytokines similar to those reported previously in gingival and periodontal fibroblasts [38, 54, 55]. hPLFs, hESC-Fib, and hESC-MSCs constitutively expressed IL-6 and IL-8, and this production was not upregulated by bacterial challenge in hESC-MSCs contrary to hPLFs and hESC-Fib. The primitive state of hESC-MSCs and ineffective downstream TLR signaling could possibly offer lower reactivity to TLR ligands and superior immunomodulatory profile in the context of bacterial infections.

Based on the differences in the structure of lipopolysaccharides, seven different serotypes (a–g) of* A. actinomycetemcomitans* are described [56]. The virulence potential of this bacterium appears to vary among different serotypes and certain serotypes/clonal types are associated with aggressive forms of periodontitis. The distribution patterns of different serotypes of* A. actinomycetemcomitans* vary among subjects of different race, ethnicity, and geographic regions [57]. In general, serotypes a–c are more prevalent among oral isolates than serotypes d–f. Further, serotype b appears to be associated with periodontal disease, while serotypes a and c are associated with periodontal health [56, 57]. In particular, serotype b JP2 clone is strongly associated with aggressive periodontitis [58, 59]. Among Asians, serotype c is more prevalent in periodontitis patients than serotype b, while among the Caucasians the serotype prevalence is the opposite [57]. A previous* in vitro* study using human gingival fibroblasts has demonstrated similar serotype-dependent differences in cytokine production [37].* A. actinomycetemcomitans* serotypes a and c were less inductive in stimulating IL-6 production while they were more inductive in IL-8 production compared to serotype b (JP2 clone). All the three* A. actinomycetemcomitans* serotypes had no significant differential effect on IL-1*β* synthesis. In this* in vitro* study, we observed that* A. actinomycetemcomitans* serotype c induced significant upregulation of* IL-1β*,* IL-6*, and* IL-8* compared to* A. actinomycetemcomitans* serotype b in hPLFs. However, exposure of hESC-Fib to the* A. actinomycetemcomitans* serotype c was associated with significant upregulation and production of IL-8 compared to serotype b but had no differential effect on IL-1*β* and IL-6 expression. Further, hESC-MSCs had no differential effect on all the three cytokines. In addition to the serotype-dependent differences in the impending bacteria, the response to bacterial challenge seems to be dependent on the cell type involved in the immune response process.

Innate immune response to bacterial challenge has different implications for different cell lineages depending on their role against pathogens. For instance, it is essential for immune cells and ECs to sense danger signals as a part of innate immune surveillance. Hence, for cellular therapy with these cells types as either primary cells or those derived from stem cells, it would be advantageous for these cells to express a functional innate immune response to bacterial challenge [45]. However, for cells like fibroblasts and MSCs which are not directly involved in immune surveillance, the insensitivity to bacterial challenge could have alternate implications. In cellular therapy related applications, in particular periodontitis, the transplanted cells will encounter bacterial challenge, hypoxia, and inflammation. The response of these cells in this microenvironment would be crucial for both initial survival of the transplanted cells and their role in tissue regeneration. For instance, lack of TLR-2 and TLR-4 mediated responses in transplanted cardiomyocytes is predicted to increase their survival, albeit retaining their response to inflammatory cytokines in the infarcted heart [60]. Similarly,* in vitro* studies have shown that hypoxia enhances the LPS-induced inflammatory cytokine expression in human periodontal ligament cells [61, 62]. Hence, the predominantly nonresponsive behaviour of hESC-MSCs to bacterial challenge found in this study could be advantageous for their survival and probably in the longer term for optimal tissue regeneration.

## 5. Conclusion

In summary, we have shown for the first time that hESC-derived progenies have phenotype-dependent response to bacterial challenge. hESC-Fib respond to bacteria through upregulation of TLRs and cytokine release, while hESC-MSCs are largely nonresponsive in spite of their constitutive TLR expression. Hence, hESC-MSCs are a promising candidate for modulating immune response in periodontitis that could influence a superior regenerative potential. Future studies on the multilineage differentiation capacity of hESC-MSCs in the presence of bacterial challenge and* in vivo* transplantation studies are needed to validate their regenerative potential.

## Figures and Tables

**Table 1:** Primer sequences used for real-time RT-PCR.

Gene	Description	Primer sequence	Product length
*β-ACTIN*	Actin, beta	F: CCAAGGCCAACCGCGAGAAGATGAC R: AGGGTACATGGTGGTGCCGCCAGAC	587 bp

*OCT4*	Octamer-binding transcription factor 4; POU class 5 homeobox 1 (POU5F1)	F: CGTGAAGCTGGAGAAGGAGAAGCTG R: AAGGGCCGCAGCTTACACATGTTC	247 bp

*SOX2*	SRY- (sex determining region Y-) Box 9	F: CCGCATGTACAACATGATGG R: CTTCTTCATGAGCGTCTTGG	370 bp

*P4Hβ*	Prolyl- 4-hydroxylase, beta subunit	F: GTCTTTGTGGAGTTCTATGCCC R: GTCATCGTCTTCCTCCATGTCT	338 bp

*COL1A1*	Collagen type I, alpha-1	F: GAACGCGTGTCAATCCCTTGT R: GAACGAGGTAGTCTTTCAGCAACA	91 bp

*COL3A1*	Collagen type III, alpha-1	F: AACACGCAAGGCTGTGAGACT R: GCCAACGTCCACACCAAATT	88 bp

*RUNX2*	Runt-related transcription factor 2	F: TGAGAGCCGCTTCTCCAACC R: GCGGAAGCATTCTGGAAGGA	266 bp

*IL-1β*	Interleukin-1-beta	F: AAGCTGAGGAAGATGCTG R: ATCTACACTCTCCAGCTG	390 bp

*IL-6*	Interleukin-6 (interferon, beta 2)	F: TGCGTCCGTAGTTTCCTTCT R: GCCTCAGACATCTCCAGTCC	141 bp

*IL-8*	Interleukin-8	F: GGTGCAGTTTTGCCAAGGAG R: TTCCTTGGGGTCCAGACAGA	183 bp

*OPG*	Osteoprotegerin	F: GCCTGGCACCAAAGTAAACG R: TACGAAGCTGCTCGAAGGTG	209 bp

*Osterix*	Osterix, transcription factor Sp7	F: CTCTGGAGTCAGAGTAGGACTGT R: CAAGGAGCCAGGCAGATGGA	197 bp

*TLR-2*	Toll-like receptor-2	F: GCCTCTCCAAGGAAGAATCC R: TCCTGTTGTTGGACAGGTCA	144 bp

*TLR-4*	Toll-like receptor-4	F: GGCAGCTCTTGGTGGAAGTT R: ACAAGCACACTGAGGACCGA	136 bp

*NANOG*	—	F: TGATTTGTGGGCCTGAAGAAAA R: GAGGCATCTCAGCAGAAGACA	60 bp

*VIMENTIN*	—	F: AGTCCACTGAGTACCGGAGAC R: CATTTCACGCATCTGGCGTTC	98 bp

## Abbreviations

*A. actinomycetemcomitans* (Aa): * Aggregatibacter actinomycetemcomitans*
EB: Embryoid body
hESC-Fib: Human embryonic stem cell-derived fibroblast-like cells
hESC-MSCs: Human embryonic stem cell-derived mesenchymal stem cells-like cells
hESCs: Human embryonic stem cells
IL: Interleukins
LPS: Lipopolysaccharides
MOI: Multiplicity of infection
MSCs: Mesenchymal stem cells
*P. gingivalis* (Pg): * Porphyromonas gingivalis*
TLR: Toll-like receptors.
